# Prolactin-induced neuroprotection against glutamate excitotoxicity is mediated by the reduction of [Ca^2+^]i overload and NF-κB activation

**DOI:** 10.1371/journal.pone.0176910

**Published:** 2017-05-05

**Authors:** Nadia A. Rivero-Segura, Edgar Flores-Soto, Selene García de la Cadena, Isabel Coronado-Mares, Juan C. Gomez-Verjan, Diana G. Ferreira, Erika Alejandra Cabrera-Reyes, Luísa V. Lopes, Lourdes Massieu, Marco Cerbón

**Affiliations:** 1 Unidad de Investigación en Reproducción Humana, Instituto Nacional de Perinatología-Facultad de Química, Universidad Nacional Autónoma de México (UNAM), Ciudad de México, México; 2 Facultad de Medicina, Dpto. de Farmacología, Universidad Nacional Autónoma de México (UNAM), Ciudad de México, México; 3 División de Neurociencias, Instituto de Fisiología Celular, Universidad Nacional Autónoma de México (UNAM), Ciudad de México, México; 4 Departamento de Investigación Básica, Instituto Nacional de Geriatría, Ciudad de México, México; 5 Instituto de Medicina Molecular, Faculdade de Medicina de Lisboa, Universidade de Lisboa, Lisboa, Portugal; Institut d'Investigacions Biomediques de Barcelona, SPAIN

## Abstract

Prolactin (PRL) is a peptidic hormone that displays pleiotropic functions in the organism including different actions in the brain. PRL exerts a neuroprotective effect against excitotoxicity produced by glutamate (Glu) or kainic acid in both *in vitro* and *in vivo* models. It is well known that Glu excitotoxicity causes cell death through apoptotic or necrotic pathways due to intracellular calcium ([Ca^2+^] i) overload. Therefore, the aim of the present study was to assess the molecular mechanisms by which PRL maintains cellular viability of primary cultures of rat hippocampal neurons exposed to Glu excitotoxicity. We determined cell viability by monitoring mitochondrial activity and using fluorescent markers for viable and dead cells. The intracellular calcium level was determined by a fluorometric assay and proteins involved in the apoptotic pathway were determined by immunoblot. Our results demonstrated that PRL afforded neuroprotection against Glu excitotoxicity, as evidenced by a decrease in propidium iodide staining and by the decrease of the LDH activity. In addition, the MTT assay shows that PRL maintains normal mitochondrial activity even in neurons exposed to Glu. Furthermore, the Glu-induced intracellular [Ca^2+^]i overload was attenuated by PRL. These data correlate with the reduction found in the level of active caspase-3 and the pro-apoptotic ratio (Bax/Bcl-2). Concomitantly, PRL elicited the nuclear translocation of the transcriptional factor NF-κB, which was detected by immunofluorescence and confocal microscopy. To our knowledge, this is the first report demonstrating that PRL prevents Glu excitotoxicity by a mechanism involving the restoration of the intracellular calcium homeostasis and mitochondrial activity, as well as an anti-apoptotic action possibly mediated by the activity of NF-κB. Overall, the current results suggest that PRL could be of potential therapeutic advantage in the treatment of neurodegenerative diseases.

## Introduction

It is well known that glutamate (Glu) is the main excitatory neurotransmitter in the central nervous system, and that excitotoxicity is induced by the sustained stimulation of glutamatergic receptors, which include: N-methyl-D-aspartic acid (NMDA), α-amino-3-hydroxy-5-methylisoxazole-4-propionate (AMPA) and kainic acid (KA) receptors [1].

Glu excitotoxicity causes neuronal cell death through the disruption of intracellular calcium ([Ca^2+^]i) homeostasis, which is followed by mitochondrial uncoupling and activation of the intrinsic mitochondrial apoptosis pathway by triggering caspases activation [2]. This condition is one of the main characteristics of many neurodegenerative disorders, including, ischemic injury, epilepsy, traumatic brain injury and neurodegenerative diseases such as Alzheimer´s and Parkinson´s [3,4].

It has been established that prolactin (PRL), a peptidic hormone mainly synthesized by lactotrophs in the anterior pituitary gland [5,6], exerts both behavioral and molecular effects in the brain. Examples of the former are the induction of maternal behavior [7] and reduction of anxiety [8]. Among the latter effects are the stimulation of neurogenesis in the olfactory bulb [9], glia activation [10], remyelination of oligodendrocytes [11], and proliferation of precursor cells in the adult mouse hippocampus [12]. Both *in vivo* and *in vitro* studies by our group, have the increased evidence supporting that PRL affords neuroprotection against excitotoxicity induced by kainic acid and Glu [13–16].

We have recently demonstrated that PRL-mediated neuroprotection in hippocampal cells occurs via the activation of its receptor [15]. However, the neuroprotective mechanism of PRL against excitotoxicity has not been completely explored, although several actions have been suggested, including changes in neurotransmission, anti-apoptotic effects, and the regulation of neurotrophic factors [17].

The classical mechanism of PRL action, involves its transcriptional activity via JAK2/STAT and MAPKs. These pathways are involved in survival, metabolism and cell proliferation [6]. However, the activation of other pathways such as nuclear factor-κB (NF-κB) by PRL have been described in other tissues [18]. NF-κB is an ubiquitous transcription factor that is activated by a variety of cytokines including the tumor necrosis factor (TNF). NF-κB activation depends on the IκB proteins phosphorylation and degradation, which allow the nuclear translocation of NF-κB to exert its transcriptional activity upon anti-apoptotic genes in different cells types including neurons, thereby blocking apoptosis [19]. Similar to the neurotrophic and neuroprotective actions of PRL in the central nervous system, it has been demonstrated that the trophic factor TGF-β1 exerts neuroprotection against NMDA-induced excitotoxicity in primary cultures of hippocampal neurons. Interestingly, this effect is mediated by an increase in Bcl-2 expression through NF-κB activation [20,21].

The mechanisms mediating PRL neuroprotection against excitotoxic neuronal death requires further clarification, therefore, the aim of the present study was to evaluate the effects of PRL on different process involved in excitotoxicity, such as the loss of the intracellular Ca^2+^ homeostasis, the disruption of mitochondrial activity, caspase activation and expression of apoptotic proteins. We observed that cells exposed to Glu in the presence of PRL, preserved the intracellular Ca^2+^ homeostasis and mitochondrial activity, inhibited caspase-3 activation and reduced the apoptotic ratio (Bax/Bcl-2). Furthermore, PRL induced the nuclear translocation of NF-κB, suggesting that its anti-apoptotic effects might be mediated by the activity of this transcription factor on downstream apoptotic genes.

## Materials and methods

### Animals

All experiments were performed in strict adherence with the international rules set out in the National Institute of Health Guide for the Care and Use of Laboratory Animals (NIH publication N0. 80–23 revised in 1996). The current protocol (LMT01-14) was approved by the Animal Care committee (CICUAL) of the Instituto de Fisiología Celular-Universidad Nacional Autónoma de México. A total of forty pregnant female rats (Wistar) were used in the experiments. The animals were kept under standard conditions in ventilated cages (12 h light/dark and 27±2°C) and fed *ad libitum* with RatChow^™^. All animals were euthanized by decapitation after deep halothane anesthesia.

### Hippocampal primary neuronal cultures

Hippocampal primary neuronal cultures were prepared from Wistar rat embryos of 17–18 days of gestation, as previously described by García de la Cadena *et al*. [22]. Cells were suspended in Neurobasal medium supplemented with 1% of B-27, 1% of B-27 minus Anti-Oxidants, 20μg/mL gentamicin (Gibco Life Technologies, Grand Island, USA) and 0.5 mM L-Glutamine (Sigma-Aldrich, St. Louis, MO, USA). Cells were cultured at a density of 2.9x10^5^ cells/cm^2^ in 35 mm Petri dishes for protein isolation or in 24-well plates pre-coated with poly-L-Lys (5 μg/mL) for viability assays and intracellular calcium concentration determinations. Cultures were incubated at 37°C in a humidified 5% CO_2_ / 95% air atmosphere until they were used for 8–10 days *in vitro* (DIV). Cells were treated with cytosine arabinoside (0.8 μM) at 4 DIV and 500 μL of fresh Neurobasal medium was added.

### Cell treatment

At 8 DIV, all cells received a partial replacement of 500 μL of Neurobasal medium and some cultures were treated with PRL (10 ng/mL) from sheep pituitary (L6520-1000IU, Sigma-Aldrich, St. Louis, USA) solubilized in sterile saline solution during 72 h. Cultures pretreated with PRL were exposed to Glu (100 μM) at 10 DIV for 24 h. Others cultures were treated only with PRL for 72 h beginning at 8 DIV, or only Glu for 24 h at 10 DIV. As a control, sterile saline solution was added to cultures during 72 h beginning at 8 DIV. Afterwards, cells were used for protein extraction, cell viability or calcium levels measurements. The doses and exposure times for PRL and Glu were chosen based on a previous study by Vergara-Castañeda *et al*. [15]

### Cell viability

Cell survival was evaluated by Syto-13/Propidium iodide (PI) fluorescent markers for viable and dead cells, as previously described by Valadas *et al*. [23]. Neurons were washed with KREBS-HEPES buffer (NaCl 117 mM, KCl 3 mM, NaHCO_3_ 26 mM, CaCl_2_ 2 mM, Glucose 10 mM, HEPES 10 mM and MgCl_2_ 1mM, pH 7.4) and incubated in the presence of Syto-13 (4 μM, emitting at 506nm when excited at 488nm) and PI (5 μg/mL; absorbing preferentially at 538 nm and emitting at 617 nm). Cells were observed by epifluorescence microscopy (Leica DM2500, Wetzlar, Germany). The data were analyzed by ImageJ free software. Cell survival is expressed as percent of positive Syto-13 neurons compared to control values; the latter was normalized to 100%.

### LDH activity determination

We evaluated the predominant cell death type in our cultures by LDH release assessment as previously reported by Páramo et al., [24]. Briefly, LDH activity was determinate in the culture medium by measuring the decrease in optical density resulting from the oxidation of NADH using pyruvate as a substrate, at 350 nm, described elsewhere [25]. The data are expressed as the difference between the initial and the final absorbance (Δ Abs).

### Mitochondrial activity determination

Mitochondrial function was determined by the MTT (3-(4,5-dimethylthiazol-2-yl)-2,5-diphenyltetrazolium) assay as previously described [26]. MTT is actively metabolized to formazan by healthy mitochondria. The absorbance of the formazan salt was read at 570nm on a plate reader.

### Intracellular calcium concentration assay ([Ca^2+^]i)

Intracellular calcium concentration was measured with a D-104 microphotometer (Photon Technology International, Princeton, NJ, USA), as previously described by Flores-Soto *et al*. [27]. At 11 DIV cells were loaded with Fura 2-AM (2.5 μM, Thermo Fisher Scientific, MA, USA) in a low concentration of Ca^2+^ (0.1 mM) and at room temperature. They were then incubated during 1 h at 37°C and under a of 5%CO_2_/95% air atmosphere. Afterwards, cells were transferred to a heated perfusion chamber mounted on an inverted Nikon Diaphot 200 microscope (Nikon, Tokyo, Japan). Cells were recorded under continuous perfusion and carbogen bubbling (to maintain pH at 7–4) at a rate of 2–2.5 mL/min with Ringer-Krebs buffer (NaCl 118 mM, NaHCO_3_ 25 mM, KCl 4.7 mM, KH_2_PO_4_ 1.2 mM, MgSO_3_ 1.2 mM, Glucose 11 mM and CaCl_2_ 2.5 mM) at 37°C. After the recording of basal fluorescence, cells were exposed to PRL (10 ng/mL) or Glu (100 μM). Cells pre-treated with PRL for 72 h were also exposed to Glu.

Neurons loaded with Fura 2-AM were alternately submitted to Xe lamp at 340 nm and 380 nm excitation light, and the emission fluorescence was measured at 510 nm. Fluorescence was measured at intervals of 0.5 s during 10 min and the intracellular Ca^2+^ concentration ([Ca^2+^]_i_) was calculated according to the Grynkiewicz formula as follows:

$${\left[Ca^{2+}\right]}_{i}=b\cdot Kd\left(R-R_{min}\right)/\left(R_{max}-R\right)$$

Where Kd is the dissociation constant of Fura-2AM, b is the ratio of fluorescent signals (R) at 380 nm for Ca^2+^-free and Ca^2+^-saturated dye, R_min_ is R in the absence of external Ca^2+^, and R_max_ is R in saturating [Ca^2+^]_i_ [28]. These parameters were determined *in vitro*. The mean 340-380nm fluorescence ratios for R_max_ (6.06) and R_min_ (0.39) were obtained by exposing the cells to Ca^2+^ (10 mM) in the presence of ionomycin (10 μM) and Ca^2+^ free Krebs with EGTA (10 mM), respectively. The fluorescence ratio at 380 nm light excitation in Ca^2+^ free medium and Ca^2+^ saturated cells was 4.23. The Kd of Fura 2-AM was assumed to be 386 nM [29].

### Western blotting

After the different treatments, cells cultured in 35 mm dishes were washed with cold PBS and lysed in RIPA lysis buffer (PBS 1x pH 7.2, 1%IGEPAL NP40, 0.1% SDS and 0.05% sodium deoxycholate) (Sigma-Aldrich, St. Louis, MO, USA) supplemented with 5 mM of a protease inhibitor cocktail (mini Complete, Roche, Mannheim, Germany). Protein concentration was determined by the Lowry method (DC protein assay, BioRad, CA, USA) [30] and 40 μg of protein sample were re-suspended in loading buffer (5% β-mercaptoethanol, 0.1% glycerol and 0.01% Bromophenol blue) (BioRad, CA, USA) and loaded onto 10% SDS-polyacrylamide gel. Protein separation was performed under denaturing conditions. Proteins were transferred to PDVF membranes by electrophoresis as previously described by Gallardo-Pérez *et al* [31]. Membranes were blocked with TBS 1X buffer containing 5% nonfat dry milk and 1% Tween-20 for 1 h at room temperature and then incubated overnight at 4°C in blocking buffer containing polyclonal primary antibodies against: anti-Bax (Cat # sc-7480 RRID:AB_626729), anti-Bcl-2 (Cat # sc-7382 RRID:AB_626736), anti-Caspase-3 (GeneTex Cat# GTX22302 RRID:AB_384753), anti-GAPDH (Cat# sc-25778, RRID:AB_10167668), or anti-β-actin (Cat # sc-47778, RRID:AB_626632). The hybridized proteins were incubated with the corresponding secondary antibodies: anti-goat (Cat # sc-2033, RRID:AB_631729), anti-mouse (Cat # sc-2302, RRID:AB_650499) and or anti-rabbit (Cat # sc-2301, RRID:AB_650500) (Santa Cruz Biotechnology, CA, USA), conjugated with horseradish peroxidase. The signal was detected by chemiluminescence using the ECL-Plus detection system (Millipore Corporation, MA, USA) with a blot-scanner (LI-COR, Lincoln, NE, USA). Densitometry analysis was performed with the Image Studio Lite version 3.1 (RRID:SCR_013715). Protein content was normalized against β-Actin or GAPDH. Control values were normalized to 100% and the density of the bands are expressed as percent of control values.

### Immunocytochemistry (ICC)

We performed the ICC for all the treatments at 11DIV. Neurons cultured in glass-rounded coverslips were washed using PBS 1X and fixed with PFA (4%). Then neurons were permeabilized with 0.05% Triton X-100 and later incubated with a polyclonal antibody anti-NF-κB (1:100, Cat. sc-372, Santa Cruz Biotechnology, CA, USA) overnight at 4°C. The hybridized cells were incubated with an anti-rabbit antibody conjugated with Alexa fluor 568 (1:500, Invitrogen). We used Alexa Fluor Phalloidin 488 (165 nM, Cat. A12379, Thermo Fisher Scientific, Waltham, MA, USA) for actin labeling and Hoechst 33342 (12μg/mL, Cat. 62249,Thermo Fisher Scientific, Waltham, MA, USA) for nucleus labeling [32].

### Imaging and 3D reconstruction of NF-κB nuclear translocation

NF-κB was detected by confocal microscopy performed at the Bioimaging Unit of the Instituto de Medicina Molecular (iMM-Lisboa, Lisboa, Portugal). Images were taken of neurons cultured in round-glass coverslips were taken using a confocal laser point-scanning Zeiss microscope LSM 880, using a 63X oil immersion objective with an Argon laser for Hoechst (RRID:AB_10626776) and Alexa Fluor Phalloidin 488, and a DPDD 562–20 nm laser for Alexa Fluor 568.

For the 3D reconstruction, confocal images were processed by Imaris for Cell Biologists (BITPLANE, UK) Software at the iMM-Lisboa and converted into a video (Supporting information).

#### Statistical analysis

All data were analyzed by Graphpad Prism^®^ software (RRID: SCR_002798) with one-way ANOVA to compare the effect between groups, followed by Tukey´s *post hoc* to find intergroup differences. Values are expressed as the mean ± SD. A statistical significance was considered at p<0.05.

## Results

### PRL prevents cell death and mitochondrial dysfunction caused by Glu-induced excitotoxicity in hippocampal neuronal cultures

**Fig 1:**
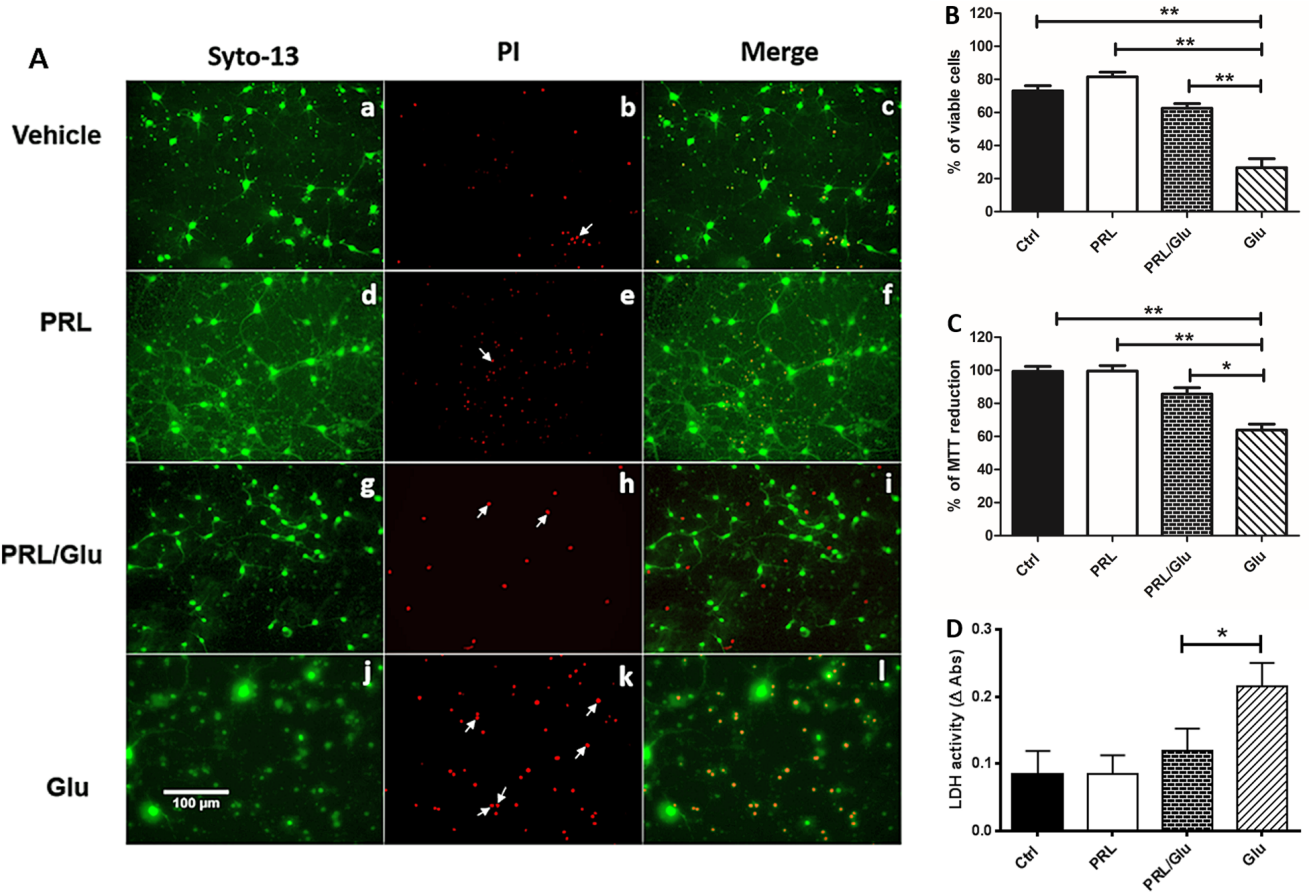
Neuroprotective effect of PRL against glutamate-induced excitotoxicity. Cell viability was assessed by the Syto-13 and propidium iodide (PI) assay and mitochondrial activity was assessed by the MTT reduction assay. Cell cultures were treated with PRL or Glu alone, or both PRL (10 ng/mL for 72 h) and Glu (100 μM for 24 h). (**A**) Representative images from neurons stained with Syto-13 (green) and PI (red) in cultures exposed to the different treatments: ***a-c*,** Vehicle (saline solution). ***d-f*,** PRL (10 ng/mL). ***g-i*,** PRL/Glu (10ng/mL and100 μM respectively), ***j-l*,** Glu (100 μM). White arrows indicate red condensed nuclei indicative of dead cells. (**B**) Values are the mean ± SD (n = 4 independent experiments). (**C**) Mitochondrial activity was assessed by MTT reduction. (**D**) LDH activity in medium culture expressed by the Δ Abs at 340nm. Data were analyzed by one-way ANOVA followed by a Tukey´s post hoc test *p<0.05 vs Glu, **p<0.001 vs Glu. Scale bar = 100 μm.

Neuroprotection induced by PRL, was evaluated using Syto-13/ PI staining, as is depicted in Fig 1(A) and 1(B). As it can be observed, PRL alone maintained neuronal viability as many Syto-13 labeled neurons are present (Fig 1A, panels d-f and Fig 1B) and neuronal morphology was preserved similar to control cultures (Fig 1A, panels a-f). In contrast, Glu exposure elicited an increase in PI-positive cells and a concomitant significant decrease in the number of Syto-13 labeled viable cells (Fig 1A, panels j-I and Fig 1B). Interestingly, combined PRL/Glu treatment improved cell viability as compared to cultures exposed only to Glu (Fig 1A, panels g-i and Fig 1B).

It is well known that excitotoxicity leads to mitochondrial dysfunction. Therefore, we explored whether PRL preserved mitochondrial activity. According to the MTT assay (Fig 1C) mitochondrial activity was indeed preserved in PRL/Glu treated neurons, thus preventing mitochondrial dysfunction. As expected, Glu induced a significant decrease in mitochondrial activity (Fig 1C).

Since it is well known that Glu treatment induces both necrotic and apoptotic cell death, we determined the LDH activity in the culture medium at the 11DIV. We observed that PRL treatment did not induce a significant LDH release *per se* and importantly reduces the Δ Abs from those cultures treated simultaneously with PRL/Glu in comparison to neurons treated with Glu alone which shows a significant increase in LDH release (Fig 1D).

### PRL inhibits the [Ca^2+^]i overload induced by Glu excitotoxicity in hippocampal neuronal cultures

**Fig 2:**
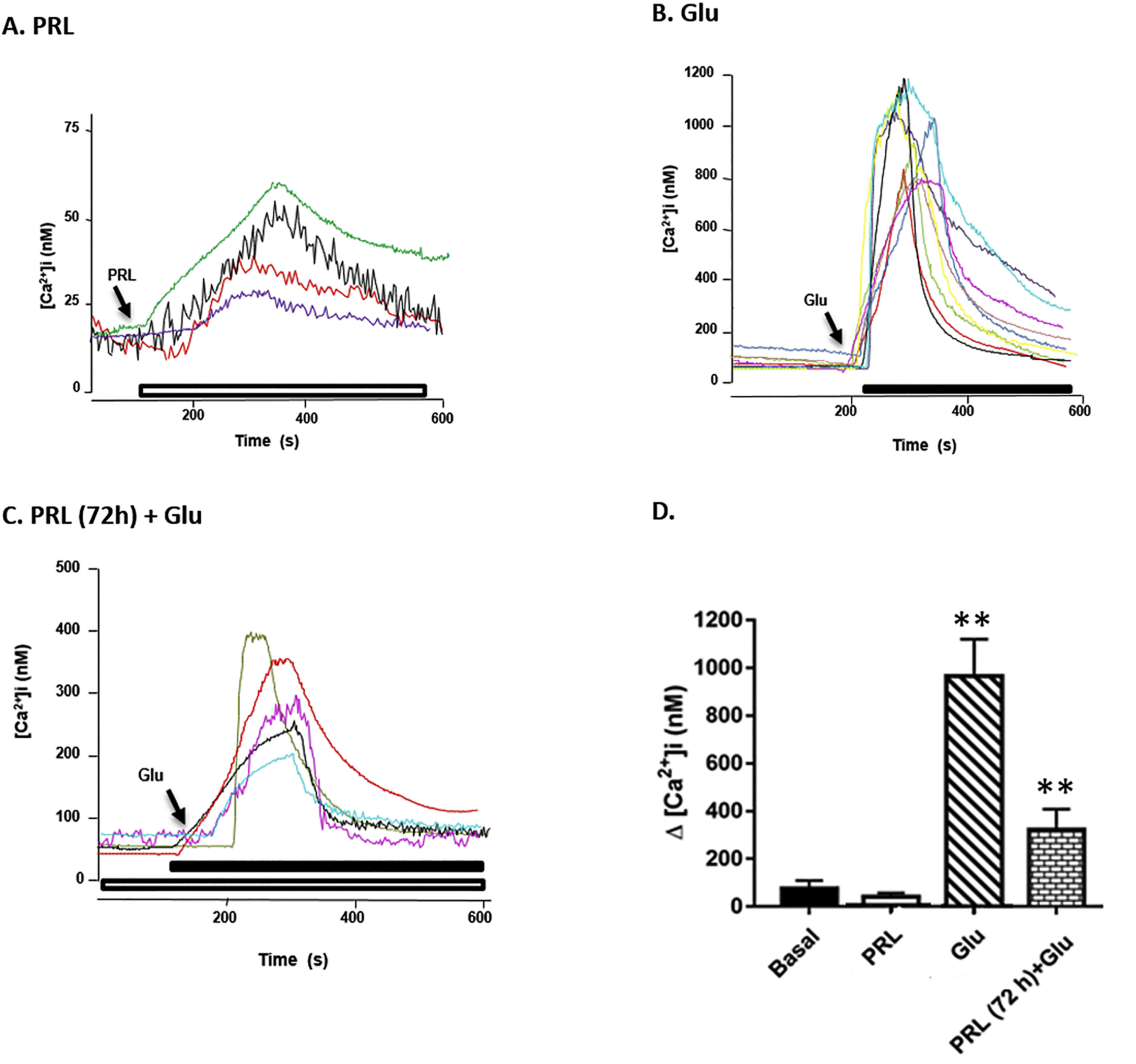
Effect of PRL on [Ca^2+^]i in a cultured of hippocampal rat neurons. (**A**) Control, neurons stimulated with PRL (10 ng/mL for 6 min). (**B**) Neurons stimulated with Glu (100 μM for 5 min). (**C**) Neurons pretreated with PRL (10 ng/mL for 72 h) and then exposed to Glu (100 μM for 6 min). Each recording of [Ca2+]i represents an independent experiment. (**D**) Bars represent the mean ± SD [Ca^2+^]i from 4–9 independent experiments. Data were analyzed by one-way ANOVA followed by Tukey´s post hoc test.** *p*<0.01 versus Glu.

One of the main triggers of excitotoxicity is [Ca^2+^]i overload, therefore, we evaluated the effect of PRL either alone or in combination with Glu on [Ca^2+^]i. Results indicate that PRL elicited a transient moderate increase in [Ca^2+^]i to 33.3±17.5 nM (Fig 2A). In contrast, cultured neurons exposed only to Glu, exhibited a significantly larger rise in [Ca^2+^]i levels (965.5±155.3 nM; Fig 2B). Meanwhile, hippocampal neurons pretreated with PRL for 72h (10 ng/mL) and then stimulated with Glu (100μM), showed a lower increase in [Ca^2+^]i levels (322.6±85.4 nM; Fig 2C and 2D), compared to Glu alone. These data are summarized in Fig 2D, which shows the [Ca^2+^]i after the different treatments.

### A PRL-induced decrease in the [Ca^2+^]i overload was associated with inhibition of procaspase-3 cleavage

**Fig 3:**
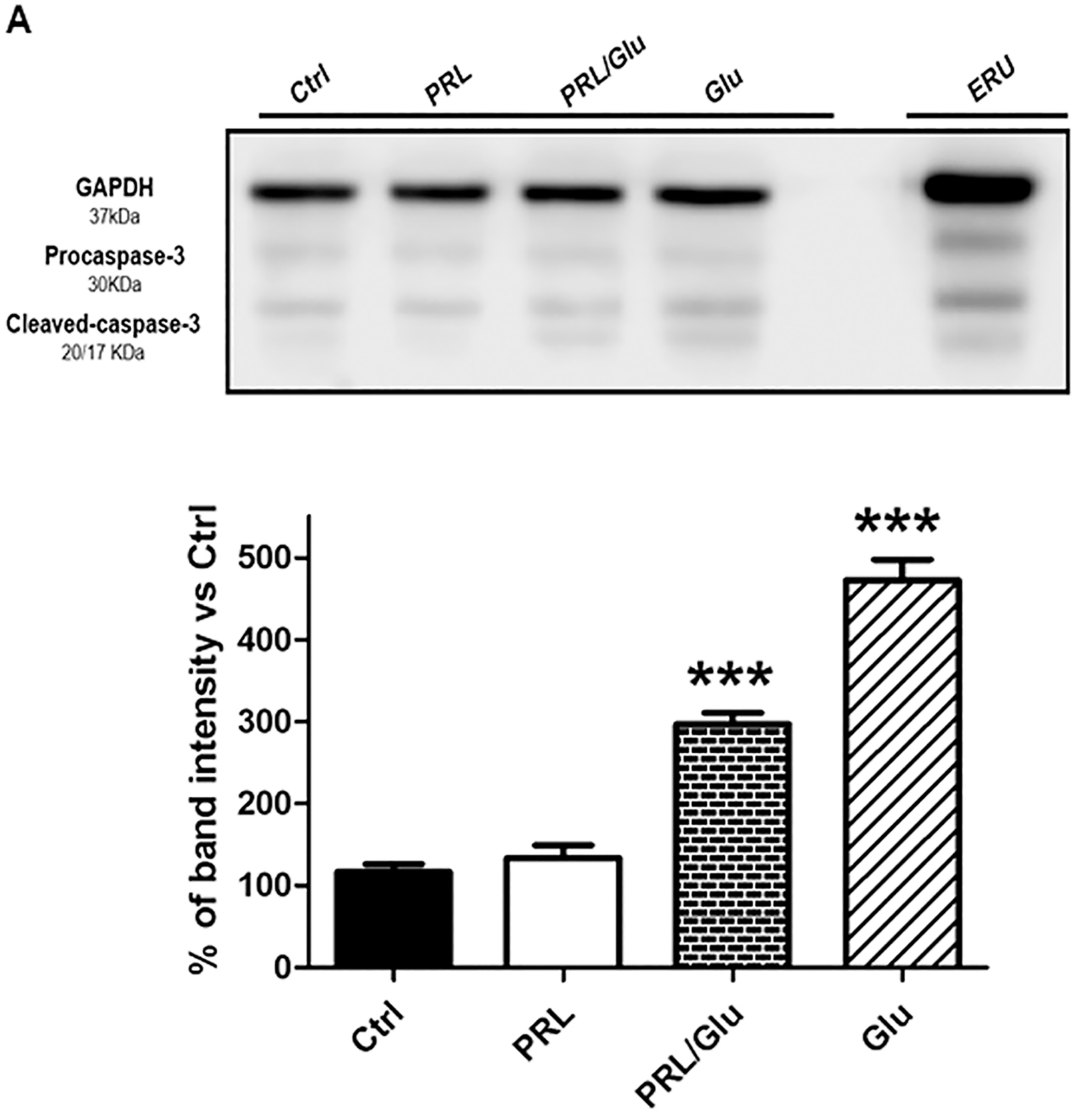
PRL prevented procaspase-3 cleavage in hippocampal neurons exposed to Glu. (**A**) Results from Western blot and densitometry analyses are expressed as the relative ratio of cleaved caspase-3/GAPDH. Bars represent the mean ± SD from 4 independent experiments. Data were analyzed by one-way ANOVA followed by Tukey´s post hoc test. ** *p*<0.01 *vs* Glu. Control (Saline Solution; Ctrl), PRL (10 ng/mL), PRL/Glu (10 ng/mL and 100 μM, respectively), Glu (100 μM), rat uterus in estrous (ERU).

It is well known that apoptotic cell death executed by procaspase-3 activation results from [Ca^2+^]i overload generated by Glu-induced excitotoxicity. Therefore, we evaluated procaspase-3 levels and its cleavage into the 20–17 kDa active fragments after the different treatments (Fig 3). As observed, the combined PRL/Glu treatment led to a significant decrease in the pro-enzyme processing and the production of its active fragment in comparison to the group treated with Glu alone. Estrous rat uterus (ERU) was used as a positive control for pro-caspase-3 processing.

### PRL promoted Bcl-2 overexpression, counteracting Glu-induced excitotoxicity

**Fig 4:**
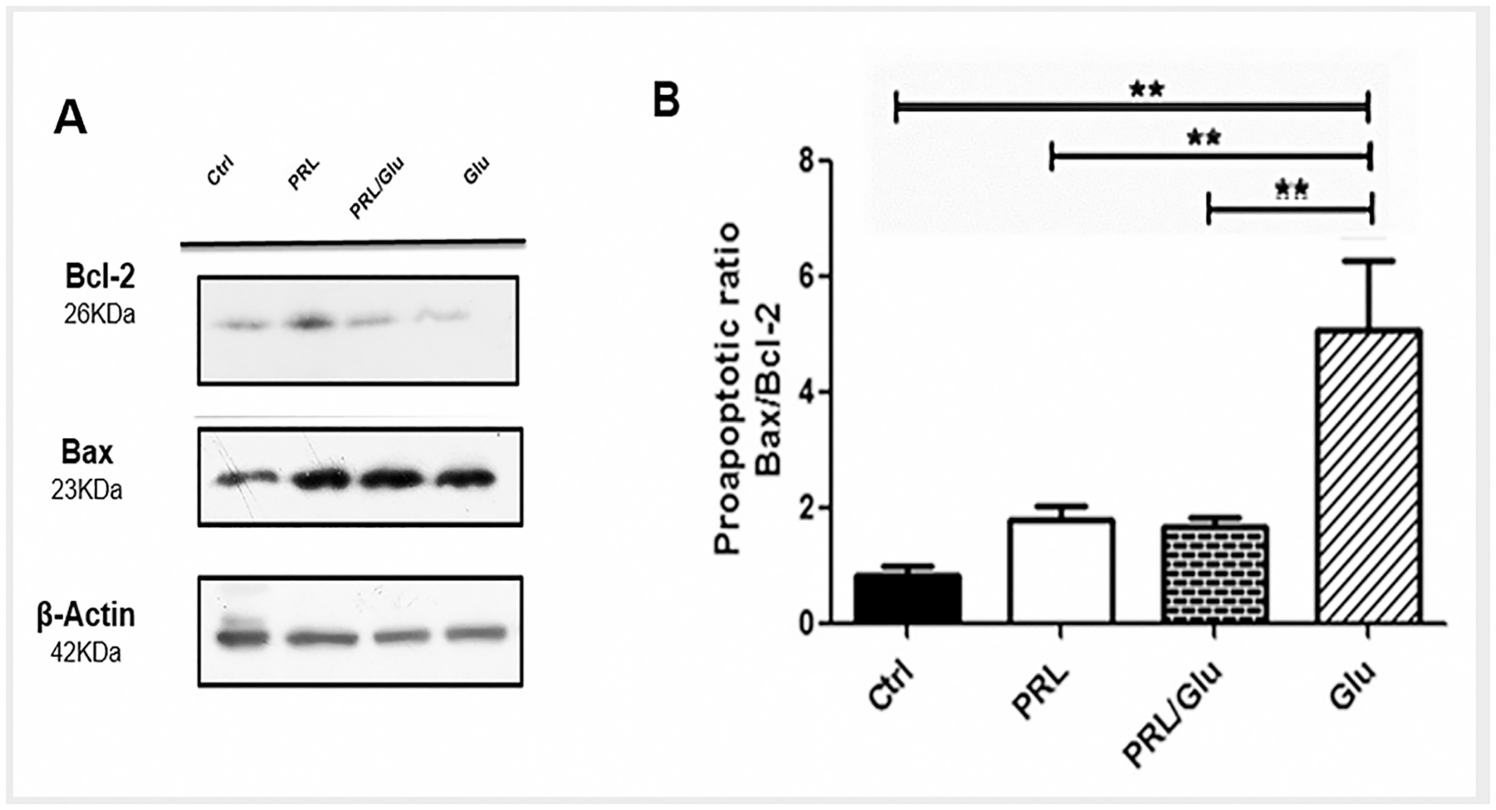
PRL decreased the proapoptotic ratio Bax/Bcl-2 in hippocampal neurons exposed with Glu. The results of Western blot and densitometry analyses are presented as the relative ratio of protein/β-actin. (**A**) Bcl-2 protein content and Bax protein content. (**B**) Pro-apoptotic ratio of Bax/Bcl-2 proteins. Bars represent the mean±SD from 4 independent experiments. Data were analyzed by one-way ANOVA followed by Tukey´s post hoc test. *p<0.05 PRL/Glu *vs* Glu, ** *p*<0.01 *vs* Glu. Control (Ctrl), PRL (10 ng/mL), PRL/Glu (10 ng/mL and 100 μM, respectively), Glu (100 μM).

The results described above indicate that improved neuronal viability by PRL treatment correlates with a reduction in Glu-induced [Ca^2+^]i raise, improved mitochondrial activity and reduced caspase-3 activation. Thus, the effect of PRL on the content of the anti- or pro-apoptotic proteins, Bcl-2 and Bax respectively, was investigated. Results indicate that PRL elicited a substantial increase in the protein content of Bcl-2 and Bax, as compared with control group, however, the pro-apoptotic ratio Bax/Bcl-2 remains close to control values. As expected, cells treated with Glu alone evoked a substantial reduction in Bcl-2 protein content and an increase in Bax levels, leading to a notable increment in the Bax/Bcl-2 pro-apoptotic ratio. Interestingly, when cultures were pretreated with PRL and exposed to Glu, the reduction in Bcl-2 content caused by Glu was completely abolished and the pro-apoptotic ratio remained as control group values (Fig 4A and 4B).

### PRL neuroprotection is associated with nuclear translocation of NF-κB

**Fig 5:**
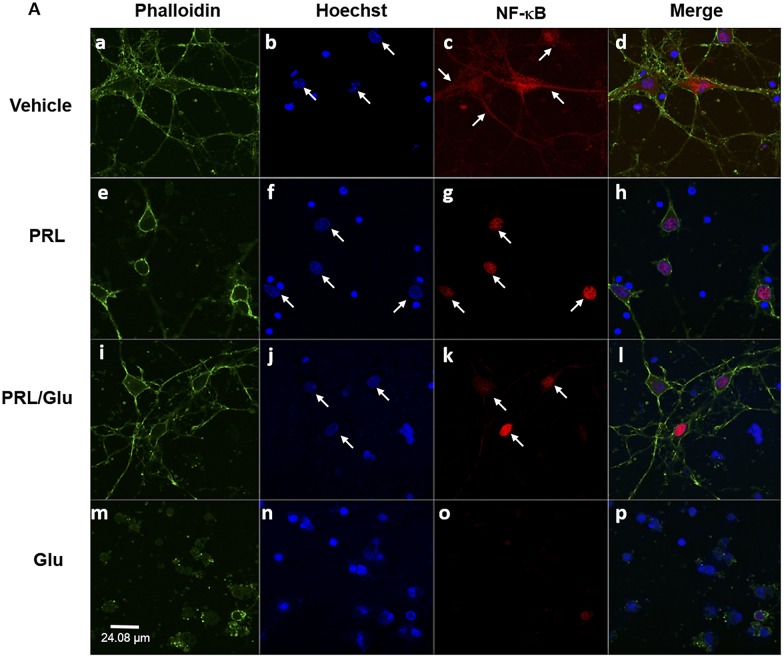
PRL induced NF-κB activation in hippocampal neurons. Nuclear translocation of NF-κB was assessed by immunochemistry. Cell cultures were exposed to PRL (10 ng/mL for 72 h) and Glu (100 μM for 24 h), or were treated with PRL or Glu alone. Representative photomicrographs from primary cultures of rat hippocampal neurons: ***a-d*,** Vehicle. ***e-h*,** PRL (10 ng/mL). ***i-l*,** PRL/Glu (10ng/mL and 100 μM respectively), ***m-p*,** Glu (100 μM). Cytoskeleton was stained green with Phalloidin 488: nuclei were stained blue with Hoechst and in NF-κB protein was labeled in red. White arrows indicate nuclear translocation of NF-κB. Scale bar = 24.08 μm.

It is well established that the Bcl-2 anti-apoptotic protein group is a direct target of the transcriptional nuclear factor NF-κB. Since PRL induces Bcl-2 overexpression in hippocampal neurons (Fig 4A and 4B), we assessed whether PRL treatment was associated with NF-κB activation. In control cultures (Ctrl) the NF-κB immune labeling signal appeared homogeneously distributed within the cell cytoplasm (Fig 5; panels a-d and S1 Video). In contrast, when neurons were treated with PRL alone, the NF-κB immune labeling signal was restricted to the nucleus (Fig 5; panels e-h and S2 Video). A similar effect was also observed in cultures treated with PRL/Glu (Fig 5; panels i-l and S3 Video). On the contrary in cells exposed to Glu alone no NF-κB signal was detected in the cytoplasm and nor in the nucleus indicating a lack of expression of the transcriptional factor after Glu treatment (Fig 5; panels m-p).

## Discussion

Excitotoxicity has been recognized as a central process in neuronal damage, such as that occurring during brain ischemia, hypoxia and neurodegenerative diseases [33]. We have previously reported, that PRL provides neuroprotection against excitotoxicity *in vivo* [13,14,16], and that it mediates (via its receptor) neuroprotection against Glu-induced excitotoxicity in rat primary cultures of hippocampal neurons [15]. In addition, other reports have also demonstrated that PRL may protect against cell damage by causing changes in neurotransmission [34,35], as well as in the regulation of neurotrophic factors and astrocyte proliferation [36]. However, the molecular mechanism by which PRL affords neuroprotection in both *in vitro* and *in vivo* models remains unclear.

The most important findings of the present study are that PRL exerts a neuroprotective action against Glu excitotoxicity through the attenuation of [Ca^2+^]i overload and the induction of NF-κB nuclear translocation. This result in a subsequent increase in Bcl-2 expression and thereby a decrease in the pro-apoptotic ratio (Bax/Bcl-2), accompanied by the preservation of mitochondrial activity and the reduction in caspase-3 activation on an *in vitro* model.

The neuroprotective action of PRL observed in the Syto-13/PI-staining and in the LDH assay presented in this study are in accordance with previous reports by our group, in which we demonstrate that PRL stimulates hippocampal neuronal survival, both in lactating rats [13,14] and in ovariectomized PRL-treated animals [16]. Interestingly, the LDH assay showed a small participation of necrotic cell death during Glu excitotoxicity as compared with the PRL effect reducing the apoptotic component of the cell death induced by Glu. The stimulatory effects of PRL on cell survival have been observed in other tissues such as, mammary epithelium during pregnancy, PRL stimulates proliferation and differentiation via the STAT5 pathway [37]. The current results also show that PRL prevents the mitochondrial dysfunction resulting from Glu-induced excitotoxity. A similar effect was observed by recombinant human growth hormone (rhGH), which prevents mitochondrial damage induced by methadone, a specific agonist of NMDA receptors [38].

Considering, that PRL maintains cell viability and mitochondrial activity, we analyzed its neuroprotective effects on intracellular [Ca^2+^] overload, which is the main factor implicated in cell death caused by excitotoxicity. It was observed that PRL treatment to hippocampal neurons evoked a transient and small increase in the [Ca^2+^]i. These data are in accordance with those of Vacher and collaborators, who found a rise in the level of [Ca^2+^]i after PRL exposure to CHO transfected cells with the long isoform of the PRL receptor [39]. Besides, it has been demonstrated that PRL induces Ca^2+^ entry and intracellular Ca^2+^ mobilization via a tyrosine kinase-dependent mechanism, demonstrating that the magnitude of PRL-induced increases in Ca^2+^ levels is enough to produce physiological responses [40].

Importantly, when neurons were pretreated with PRL during 72 h and later exposed to Glu, the increase in [Ca^2+^]i was significantly attenuated. This effect might be related to an antagonistic effect of PRL on glutamate receptors. In accordance, a recent study, showed that rhGH, a member of the growth hormone-like superfamily, which includes PRL, acted as a neuroprotector against methadone-induced toxicity in primary cultures of cortical neurons, through the alteration of the expression of NMDA receptors subunits [38]. Our group is currently exploring the possible effect of PRL on the modulation of glutamate receptors in the present neurotoxicity model.

On the other hand, it is well known that a Glu-induced [Ca^2+^]i overload leads to the activation of apoptotic cell death [41], since we demonstrated that PRL decrease both the apoptotic cell death and [Ca^2+^]i overload. Therefore, we evaluated the effect of PRL on the apoptotic caspase pathway, which depends on the cleavage of procaspase-3 into its active fragments and on changes in Bax/Bcl-2 pro-apoptotic ratio. We observed that PRL significantly reduced procaspase-3 cleavage. This observation is in agreement with previous studies, reporting that PRL down-regulates caspase-3 mRNA levels and enzyme activity in rat *decidua* [42], and that it induces a decrease in the spontaneous DNA strand breaks and the concomitant suppression of caspase activation in human spermatozoa [43]. In addition, it has been shown that PRL stimulates survival in Nb2 cells exposed to dexamethasone by blocking caspase activation and inducing Bcl-2 overexpression [44,45].

The induction of apoptosis depends on the balance between pro- and anti-apoptotic proteins more than their individual levels. The present results demonstrate that cells exposed to Glu alone showed a notable increase in the pro-apoptotic ratio (Bax/Bcl-2) due to a decrease in Bcl-2 and an increase in Bax. Despite increased Bax levels observed in the presence of PRL, the pro-apoptotic value, for neurons exposed only to PRL or with PRL/Glu, was similar to that found in the control group, due to the increment of Bcl-2 protein expression. Thus, suggesting that the neuroprotective effect of PRL may also be mediated by an increase of the anti-apoptotic protein Bcl-2. This observation that PRL improves cell viability through the overexpression of Bcl-2, has been reported in chondrocytes treated with PRL and deprived of serum or exposed to INF-γ to induce apoptosis [46,47]. Transcriptional changes in anti-apoptotic genes have been previously observed in pancreatic β-islets exposed to PRL [48,49]. In a previous study using a similar model of excitotoxic damage, the authors reported that protection by TGF-β1 against NMDA toxicity was associated with an increase in Bcl-2 and Bcl-xL anti-apoptotic proteins [50]. On the other hand, there are some reports on both in vivo and in vitro models, in which it has been reported that leptin (a member of adipocytokines like PRL) exerts neuroprotective effects through the inhibition of apoptosis via JAK/STAT pathway activation [51,52]. Moreover, these changes have been associated with NF-κB activation in hippocampal neurons treated with TGF-β1 [21,53]. These observations were similar to our present results, which showed that PRL treatment stimulated NF-κB activation, while untreated neurons lost the NF-κB signal.

In contrast, It has been reported that Bax induction in hippocampal and cortical neurons might be induced by an independent NF-kB pathway [54], and thus, further research about parallel Bcl-2 and Bax induction by PRL during neuronal excitotoxic damage requires to be clarified. Hence, these observations support the critical role of Bcl-2 expression in the neuroprotective effect of PRL against Glu-induced excitotoxicity.

## Conclusions

**Fig 6:**
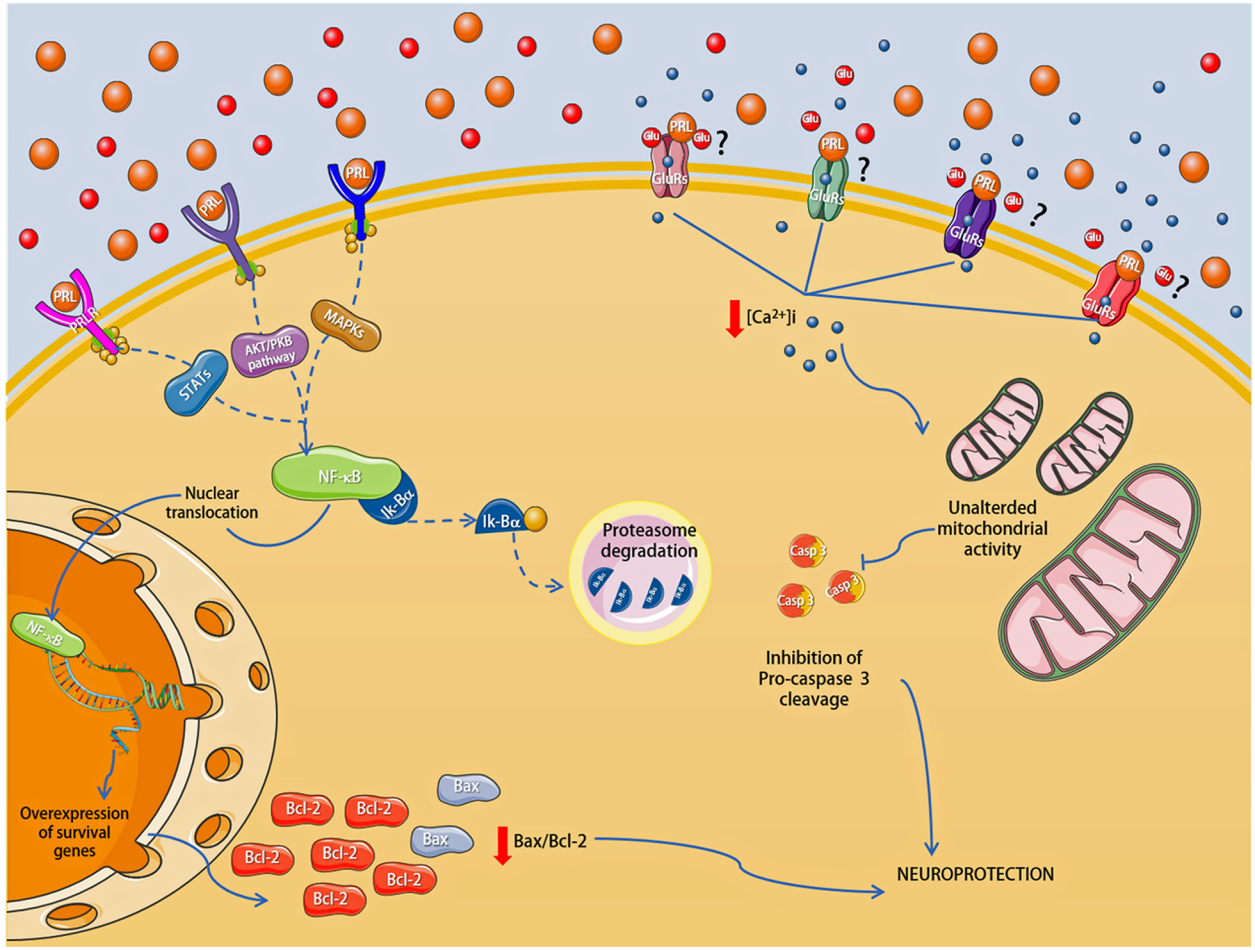
Proposed molecular mechanisms involved in the PRL-mediated neuroprotective effect against Glu-induce excitotoxicity in primary cultures of rat hippocampal neurons, which includes. **a)** Regulation of [Ca^2+^]i homeostasis, **b)** NF-κB activation and the **c)** Concomitant overexpression of anti-apoptotic proteins. Question marks and dotted lines indicate hypothetical relationships.

Overall, the current results suggest that PRL afforded neuroprotection effect against Glu-induced excitotoxicity in primary cultures of rat hippocampal neurons, due in part to the preservation of [Ca^2+^]i homeostasis, and cell survival through the up-regulation of Bcl-2 protein expression, probably via NF-κB pathway. In Fig 6 we summarized the suggested molecular mechanisms involved in PRL-induced neuroprotection, described in the present study. Finally, our results highlight the potentiality of PRL as a useful molecule for the treatment of neurodegenerative conditions and neurological diseases.

## Supporting information

S1 Video Under standard conditions NF-κB is spread in the body of the neuron. Cytoskeleton was stained green with Phalloidin 488: nuclei were stained blue with Hoechst and in NF-κB protein was labeled in red.
(MP4)

S2 Video PRL treatment induces NF-κB activation in hippocampal neurons. Cytoskeleton was stained green with Phalloidin 488: nuclei were stained blue with Hoechst and in NF-κB protein was labeled in red.
(MP4)

S3 Video Neurons treated with both PRL/Glu maintains the NF-κB activation. Cytoskeleton was stained green with Phalloidin 488: nuclei were stained blue with Hoechst and in NF-κB protein was labeled in red.
(MP4)
